# Remarkable bismuth-gold alloy decorated on MWCNT for glucose electrooxidation: the effect of bismuth promotion and optimization via response surface methodology

**DOI:** 10.3906/kim-2102-16

**Published:** 2021-08-27

**Authors:** Ömer Faruk ER, Berdan ULAŞ, Hilal DEMİR KIVRAK

**Affiliations:** 1 Department of Chemical Engineering, Faculty of Engineering, Van Yüzüncü Yıl University, Van Turkey; 2 Department of Mining Engineering, Faculty of Engineering, Van Yüzüncü Yıl University, Van Turkey; 3 Department of Chemical Engineering, Faculty of Engineering and Architectural Sciences, Eskişehir Osmangazi University, Eskişehir Turkey

**Keywords:** Gold, bismuth, carbon nanotube, glucose, electrooxidation, response surface methodology (RSM)

## Abstract

In this study, the carbon nanotube supported gold, bismuth, and gold-bismuth(Au/MWCNT, Bi/MWCNT, and Au-Bi/MWCNT) nanocatalysts were prepared with NaBH_4_ reduction method at varying molar atomic ratio for glucose electrooxidation (GAEO). The synthesized nanocatalysts at different Au: Bi atomic ratios are characterized via *x* - *ray diffraction* (XRD), transmission electron microscopy (TEM), and N_2_ adsorption-desorption. For the performance of AuBi/MWCNT for GAEO, electrochemical measurements are performed by using different electrochemical techniques namely cyclic voltammetry (CV), linear sweep voltammetry (LSV), chronoamperometry (CA), and electrochemical impedance spectroscopy (EIS). Monometallic Au/MWCNT exhibits higher activity than Bi/MWCNT with 256.57 mA/mg (0.936 mA/cm^2^) current density. According to CV results, Au_80_Bi_20_/MWCNT nanocatalyst has the highest GAEO activity with the mass activity of 320.15 mA/mg (1.133 mA/cm^2^). For Au_80_Bi_20_/MWCNT, central composite design (CCD) is utilized for optimum conditions of the electrode preparation. Au_80_Bi_20_/MWCNT nanocatalysts are promising anode nanocatalysts for direct glucose fuel cells (DGFCs).

## 1. Introduction

Energy is a vital and permanent need for human life and welfare [1–7]. Fuels such as formic acid [8–10], glucose (GA) [11], ethanol [12, 13], ethylene glycol [14], hydrazine [15], and methanol [16, 17] are principal sources. From these sources, GA is given great significance due to its advantages such as abundance in nature, cheapness, non-toxicity, and easy to transport [18–20]. As a result of complete oxidation of GA, 24 e^-^ are released [21]. However, the complete electrooxidation of GA has not been achieved yet. Complete oxidation of GA consists of a complex reaction sequence [22]. Glucose electrooxidation (GAEO) to gluconic acid reactions are given as follows [23, 24]:

Anode: C_6_H_12_O_6_ + 2OH^-^ C_6_H_12_O_7_ +H_2_O +2e^-^ (1)

Cathode: 0.5O_2_ +H_2_O +2e^-^ 2OH^-^ (2)

Overall: C_6_H_12_O_6_ + 0.5O_2_ C_6_H_12_O_7_ (3)

The development of anode catalysts with high GAEO activity is crucial for the commercialization of the direct glucose fuel cell. Hence, the anode catalyst performance of Fe_15_Pt_85_ [25], Ni-Fe [26], Ni-Co [27], Au [28], Pt [29], G-ITO [30], AgNi [31], Pd [32], FeCo_2_O_4_ [33], and Pd-Au [34] nanocatalysts have been investigated for direct GA fuel cells (DGFC). For instance, Chai et al. reported that Pd_3_Cu-B/C nanocatalyst synthesized by a simple aqueous phase approach method had high GAEO activity and stability for GAEO reaction in fuel cell [35]. Likewise, Yan et al. stated that Pd_70_Au_30_ nanocatalyst prepared via modified pulse microwave-assisted polyol method had high current density [36]. Another study performed by Chai et al. on the synthesis of Pd-SnCoO_x_/C nanocatalysts and investigation of their GAEO activities revealed that Pd-SnCoO_x_/C had enhanced activity and outstanding stability with a great active surface area compared to Pd/C nanocatalyst [37]. Related literature was given in Table 1.

**Table 1:** Maximum current densities for GAEO values reported in literature.

Nanocatalyst	Maximum peak mA/cm^2^	Reference
Pd_3_Rh/C	1.7	(38)
AuAg/C	3.75	(39)
Pd_3_Sn_2_/C	3.64	(40)
PtRu/C	2.74	(41)
PtBi/C	2.25	(42)
Cu@Cu_2_O-Pd	1.15	(43)

Herein, we aim to investigate the effect of Bi addition to Au in terms of GAEO activity. Thus, Au/MWCNT, Bi/MWCNT, and Au-Bi/MWCNT nanocatalysts were prepared via NaBH_4_ reduction method, and these nanocatalysts were characterized by XRD, BET, and TEM. To investigate the effect of Bi promotion, GAEO activities of these nanocatalysts are measured via cyclic voltammetry (CV), linear sweep voltammetry (LSV), chronoamperometry (CA), and electrochemical impedance spectroscopy (EIS). For Au_80_Bi_20_/MWCNT nanocatalyst, central composite design (CCD) was utilized for determining the optimum conditions of electrode preparation. The volume of nanocatalyst slurry (V_c_, A), ultrasonication time of the nanocatalyst slurry (t_u_, B), and the drying time of the electrode (t_d_, C) were determined as independent variables.

## 2. Materials and method

All chemicals used were purchased from Sigma-Aldrich. Au/MWCNT nanocatalysts and Bi/MWCNT nanocatalysts were synthesized by NaBH_4_ reduction method with AuCl_3_ and Bi(NO_3_)_3_.5H_2_O metal as a precursor, respectively. Metal precursors were dissolved in deionized water and mixed with 0.1 g MWCNT by adding NaBH_4_ at ratio of NaBH_4_:Au (15:1). These mixtures were filtered and washed. Likewise, Au-Bi/MWCNT nanocatalysts were prepared by NaBH_4_ reduction method at different Au:Bi ratios as 95:5, 90:10, 80:20, 70:30, 60:40, and 50:50. 

Au/MWCNT, Bi/MWCNT, and Au_80_Bi_20_/MWCNT nanocatalysts were characterized via N_2_ adsorption and desorption (Micromeritics 3Flex equipment Tristar II 3020), XRD (PANalytical Empyrean device-ray diffractometer with Cu Kα radiation (λ = 1.54056 Å)), and C-TEM (Hitachi HighTech HT7700 high re-transmission electron microscope operating at 120 kV). 

All electrochemical properties of Au/MWCNT, Bi/MWCNT, and Au-Bi/MWCNT nanocatalysts were determined by CV, LSV, CA, and EIS in 0.5 M GA. A nanocatalyst ink was obtained by dispersing 3 mg nanocatalyst in 1 mL of Nafion. Then, 5 mL of nanocatalyst ink was transferred to glassy carbon electrode and dried. CV measurements were performed at –0.6 V to 0.8 V potentials at 50 mv s^–1^ scan rate. Stability measurements were conducted by CA during 1000 s. 

CCD was utilized for optimum conditions of the electrode preparation. The volume of nanocatalyst slurry (V_c_, A), ultrasonication time of the nanocatalyst slurry (t_u_, B), and the drying time of the electrode (t_d_, C) are determined as independent variables. The maximum current density values obtained for GAEO were identified as the response. The error for the value of response was determined by 6 experiments at the middle levels of the parameters, and 20 sets of experiments were performed in total. Table 2 depicts the experimental points determined by Design Expert 7.0 and their corresponding response values, where, ˗ 1, 0, and + 1 represent the lowest, central, and highest levels of the parameters. Interactions between independent parameters were statistically evaluated with analysis of variance (ANOVA), and the suitability of the proposed model was tested with the coefficient of determination ( *R* *^2^* ). 

**Table 2:** Experimental design of CCD and obtained responses.

	A	B	C	Response
Run	Vc	tu	td	Specific activity
	µL	min	min	mA/cm2
1	7.75 (0)	30.50 (0)	30.00 (+1)	0.89
2	0.50 (–1)	30.50 (0)	15.50 (0)	1.41
3	7.75 (0)	60.00 (+1)	15.50 (0)	0.94
4	15.0 (+1)	60.00 (+1)	30.00 (+1)	0.73
5	7.75 (0)	30.50 (0)	15.50 (0)	0.96
6	15.0 (+1)	1.00 (–1)	1.00 (–1)	0.66
7	7.75 (0)	30.50 (0)	15.50 (0)	1.02
8	15.0 (+1)	1.00 (–1)	30.00 (+1)	0.79
9	15.0 (+1)	60.00 (+1)	1.00 (–1)	0.83
10	15.0 (+1)	30.50 (0)	15.50 (0)	1
11	0.50 (–1)	1.00 (–1)	30.00 (+1)	0.6
12	7.75 (0)	30.50 (0)	1.00 (–1)	0.83
13	7.75 (0)	30.50 (0)	15.50 (0)	0.97
14	7.75 (0)	1.00 (–1)	15.50 (0)	0.55
15	0.50 (–1)	1.00 (–1)	1.00 (–1)	0.86
16	7.75 (0)	30.50 (0)	15.50 (0)	1.03
17	7.75 (0)	30.50 (0)	15.50 (0)	0.95
18	0.50 (–1)	60.00 (+1)	1.00 (–1)	1.21
19	0.50 (–1)	60.00 (+1)	30.00 (+1)	1.14
20	7.75 (0)	30.50 (0)	15.50 (0)	1.17

## 3. Results and discussion

### 3.1. Characterization results

**Figure 1:**
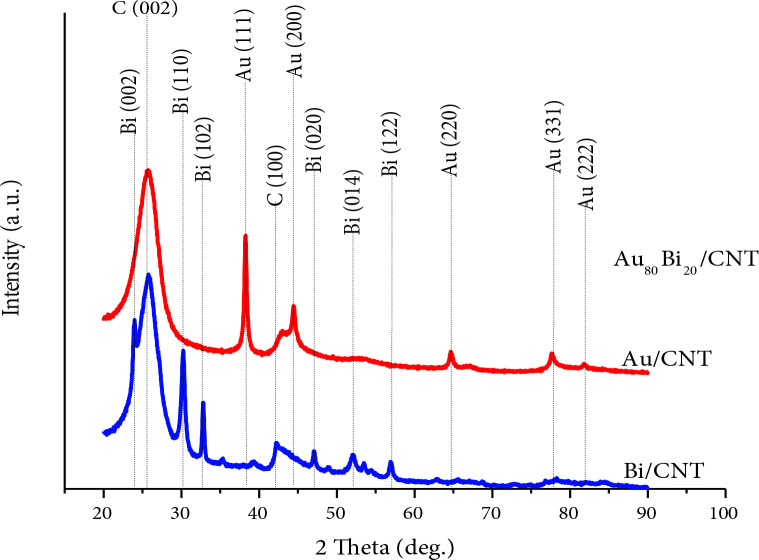
The XRD patterns of the monometallic Bi/MWCNT, Au/MWCNT, and bimetallic Au80Bi20/MWCNT nanocatalysts.

Characterizations of Au/MWCNT, Bi/MWCNT, and Au_80_Bi_20_/MWCNT nanocatalysts were performed with XRD, BET, and TEM. XRD patterns of MWCNT supported Au, Bi, and Au_80_Bi_20_ nanocatalysts were given in Figure 1. The diffraction peaks of Au and Bi were clearly seen in Figure 1. The diffraction peaks of C (0 0 2) and C (1 0 0) planes were observed at around 25.5° and 42.8° for all nanocatalysts, respectively. The presence of the C (0 0 2) indicates that the carbon in the structure is hexagonal carbon [44]. The (1 1 1), (2 0 0), (2 2 0), (3 1 1), and (2 2 2) facets of Au were obtained at 38.3°, 44.4°, 64.5°, 77.3°, and 81.9° 2θ values for Au/MWCNT. These peaks are specific crystallographic planes of the face-centered cubic (fcc) Au [45, 46] (Figure 1). The average diameter of the nanocatalysts can be achieved by using Scherrer’s Equation [47,48]. The crystal size of Au/MWCNT was found as 19.08 nm. For Bi/MWCNT, the (0 0 2), (1 1 0), (1 0 2), (0 2 0), (0 1 4), and (1 2 2) facets of Bi were observed at 24°, 30.2°, 32.9°, 46.9°, 51.8° and 56.9° 2θ values, respectively (Figure 1). As clearly seen from Figure 1, all diffraction peaks of Au and Bi were observed for Au_80_Bi_20_/MWCNT nanocatalyst. These results depict that the face-centered cubic (fcc) of Au maintains its structure and increases the GAEO activity of the nanocatalyst by increasing the number of active sites of Bi. The crystal size of Au_80_Bi_20_/MWCNT nanocatalyst was found as 21.96 nm. Moreover, the average inter-planar distances of Au/CNT, Bi/CNT, and Au_80_Bi_20_/MWCNT nanocatalysts were calculated using Bragg’s Law [49,50]. The 2θ values of Au (111) and Bi (110) peaks, which are the most intense peaks in the XRD patterns, were used. The average interplanar distance for Au/CNT, Bi/CNT, and Au_80_Bi_20_/MWCNT nanocatalysts was calculated as 2.35, 2.95, and 2.35 nm, respectively. 

**Figure 2:**
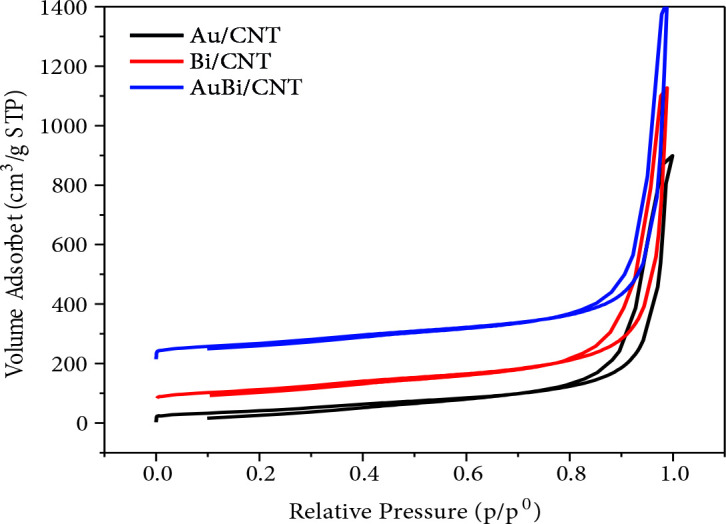
N_2_ adsorption-desorption isotherms of Au/MWCNT, Bi/MWCNT, and Au_80_Bi_20_/MWCNT nanocatalysts.

N_2_ adsorption-desorption were used to determine pore size, BET surface area, and pore volume of Au/MWCNT, Bi/MWCNT, and Au_80_Bi_20_/MWCNT. BET surface area, pore size, and pore volume of nanocatalysts were given in Figure 2 and Table 3. In this study, all of the used nanocatalysts were exhibited the V-type adsorption-desorption isotherm with H1 type hysteresis loop [51]. This indicates that the catalysts are mesoporous in according to International Union of Pure and Applied Chemistry (IUPAC) categorization. BET surface areas of Au/MWCNT, Bi/MWCNT, and Au_80_Bi_20_/MWCNT were found as 159.0, 225.1, and 221.6 m^2^/g, respectively. As can be clearly seen from Table 3, the use of Bi and Au together increased the BET surface area. Likewise, the increase in pore volume and pore size of the nanocatalyst were observed (Table 3). According to the pore size and pore volume, the nanocatalysts are sorted Au_80_Bi_20_/MWCNT > Bi/MWCNT > Au/MWCNT. 

**Table 3:** Summary of the BET of result of Au/MWCNT, Bi/MWCNT, and Au80Bi20/MWCNT.

Nanocatalyst	BET surface area (m2/g)	Pore volume (cm3/g)	Pore size (nm)
Au/MWCNT	159.0	1.23	24.5
Bi/MWCNT	225.1	1.65	25.6
Au80Bi20/MWCNT	221.6	1.86	28.7

**Figure 3:**
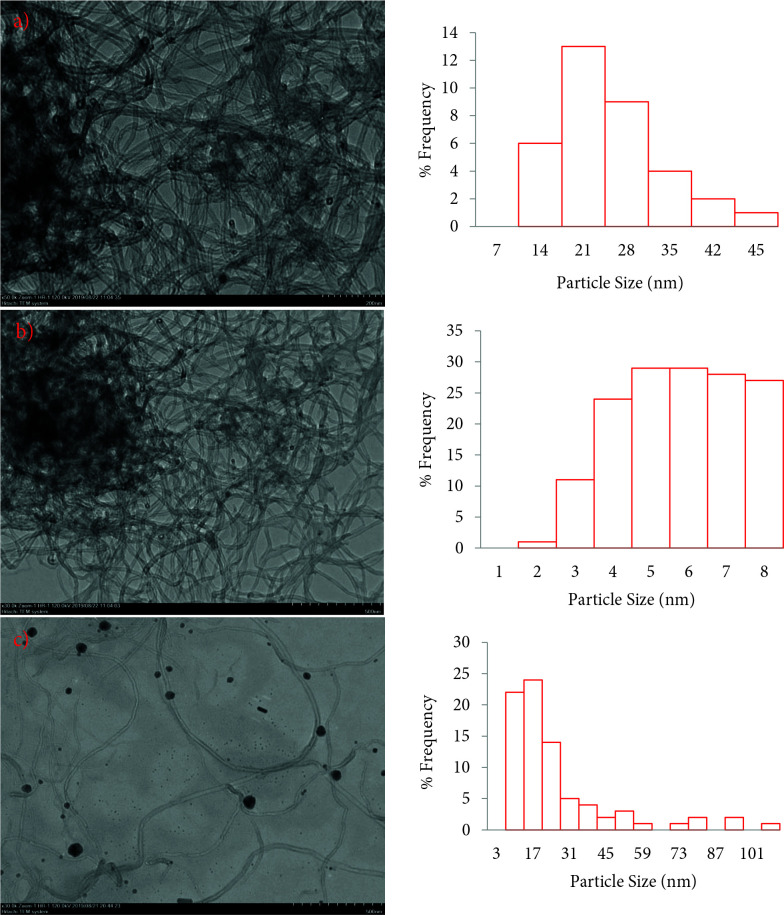

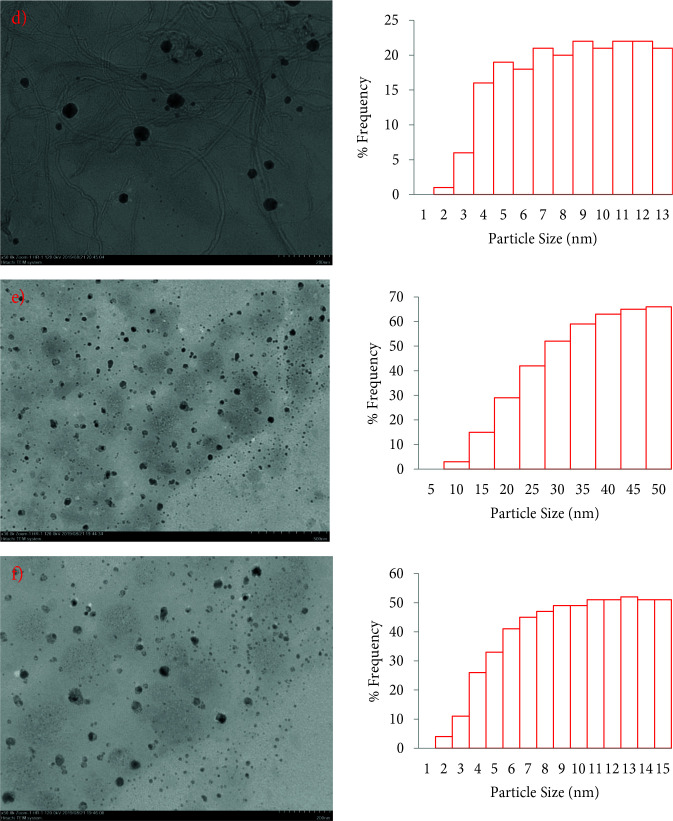
TEM images of (a and b) Au_80_Bi_20_/MWCNT, (c and d) Au/MWCNT, and (e and f) Bi/MWCNT nano-catalysts (corresponding histogram of particle size distribution).

The morphological and particle size of the Au/MWCNT, Bi/MWCNT, and Au_80_Bi_20_/MWCNT nanocatalysts was determined with TEM and were depicted in Figure 3. It is explicit that Au and Bi nanoparticles were agglomerated for Au/MWCNT and Bi/MWCNT nano-catalysts. However, it can be clearly seen that such a situation is not observed for Au_80_Bi_20_/MWCNT nanocatalyst and that there is a homogeneous distribution. This can be explained by the fact that Bi nanoparticles on the MWCNT surface have a positive effect by entering between the Au nanoparticles. The increase in BET surface area of Au_80_Bi_20_/MWCNT compared to Au/CNT could support this positive effect. The average particle size for Au/MWCNT, Bi/MWCNT, and Au_80_Bi_20_/MWCNT nanocatalysts was found as 26.8, 23.2, and 19.38 nm, respectively. The particle size for Au_80_Bi_20_/MWCNT was found to be consistent with crystal sizes obtained from the XRD result.

### 3.2. Electrochemical assessment

**Figure 4:**
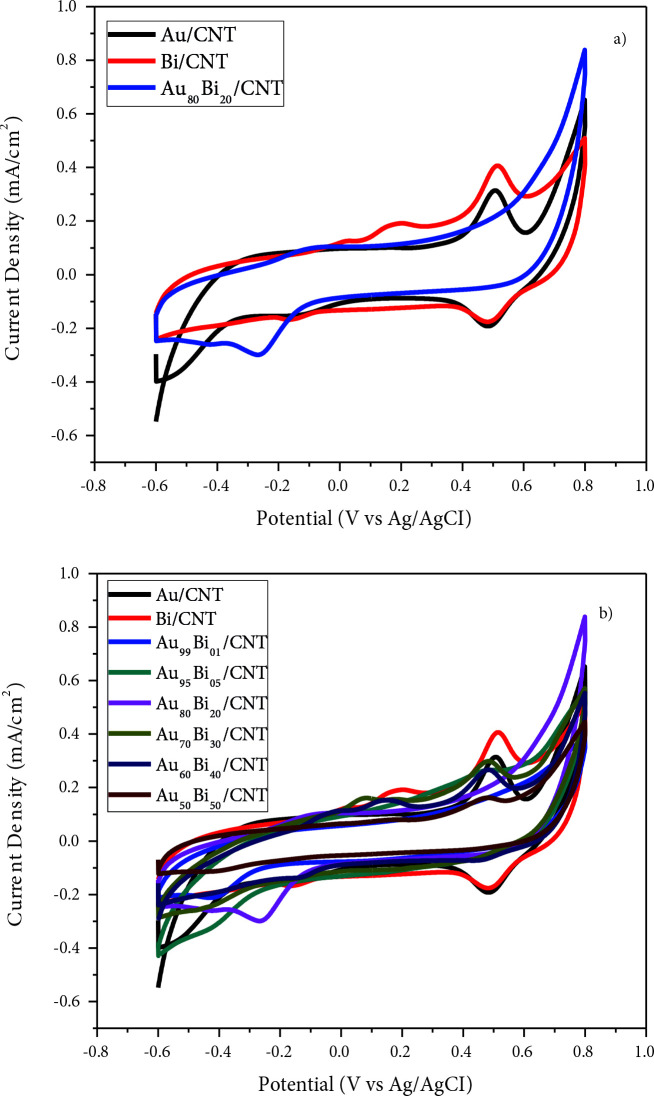
Cyclic voltammograms in 1 M KOH for electrodes modified with a) 3% Au/MWCNT, 3% Bi/MWCNT, and 3% Au_80_Bi_20_/MWCNT nanocatalysts; b) 3% Au/MWCNT, 3% Bi/MWCNT, and 3% AuBi/MWCNT nano-catalysts (scan rate: 50 mV s^-1^).

Au/MWCNT, Bi/MWCNT, and Au-Bi/MWCNT nanocatalysts were prepared via the NaBH_4_ reduction method to investigate their GAEO activity. Figure 4 depicts electrooxidation measurement of Au/MWCNT, Bi/MWCNT, and Au-Bi/MWCNT in 0.5 GA solution. Synthesized nanocatalysts were tested in 1 M KOH and 0.5 M GA, respectively. In this way, the best atomic molar ratios were determined and the second stage was passed. 

**Figure 5:**
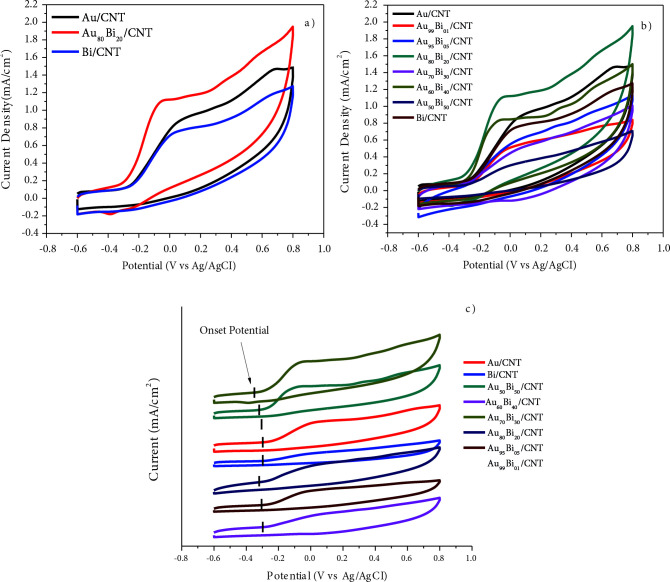
Cyclic voltammograms in 1 M KOH + 0.5 M GA for electrodes modified with a) 3% Au/MWCNT, 3% Bi/MWCNT, and 3% Au_80_Bi_20_/MWCNT nanocatalysts; b) 3% Au/MWCNT, 3% Bi/MWCNT, and 3% AuBi/MWCNT nanocatalysts; c) onset potentials of all nanocatalysts (scan rate: 50 mV s–1).

The hydroxide (OH^-^) adsorption-desorption peak was observed for Au and Bi between 0.4 V and 0.6 V, while these peaks were not visible for Au_80_Bi_20_ (Figure 4a). As described in the literature, the Au desorption peak is obtained due to the reduction of the oxidative gold layer [52]. Due to the dispersion of Bi nanoparticles in the Au layer formed on the surface of the AuBi/MWCNT nanocatalyst, it could prevent oxidation at the positive forward direction peak of the Au layer. As seen, electrooxidation peaks were obtained for all nanocatalysts prepared. When using Bi together with Au, it is observed that the current density is clearly increased. Au_80_Bi_20_/MWCNT nanocatalyst exhibited the highest performance among prepared nanocatalysts with 1.133 mA/cm^2^ (320.1 mA/mg Au) for GAEO (Figure 5 and Table 4). These results are consistent with CA and EIS measurements.

**Table 4:** Electrochemical properties of synthesized nanocatalysts for GAEO.

Nanocatalyst	Peak potential of forward peak, V	Specific activity,mA/cm2	Mass activity,mA/mg Pd	Onset potential, V
Au/MWCNT	0.103	0.936	256.5	–0.291
Bi/MWCNT	0.036	0.764	202.3	–0.307
AuBi(99:01)/MWCNT	0.016	0.519	142.3	–-0.264
AuBi(95:05)/MWCNT	0.026	0.595	159.4	–0.269
AuBi(80:20)/MWCNT	–0.039	1.133	320.1	–0.345
AuBi(70:30)/MWCNT	0.057	0.510	134.7	–0.307
AuBi(60:40)/MWCNT	–0.060	0.840	232.6	–0.309
AuBi(50:50)/MWCNT	–0.014	0.302	84.5	–0.258

**Figure 6:**
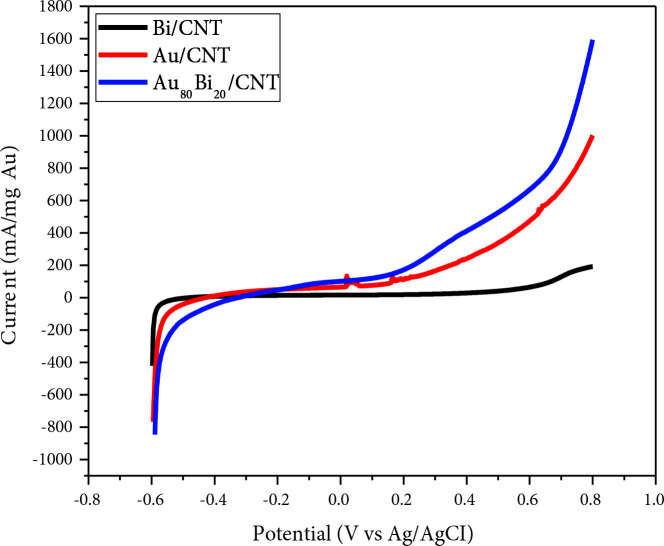
LSV profiles of 3% Au/MWCNT, 3% Bi/MWCNT, and 3% Au_80_Bi_20_/MWCNT nanocatalysts in 1 M KOH + 0.5 M GA for modified electrode.

The mass activities of Au/MWCNT, Bi/MWCNT, and Au_80_Bi_20_/MWCNT catalysts were examined via LSV technique at a scan rate of 50 mV s^–1^. LSV profile of these nanocatalysts in 1 M KOH + 0.5 M GA solution were given in Figure 6. As could be seen in Figure 6, Au_80_Bi_20_/MWCNT nanocatalyst exhibited a higher mass activity compared to these of Au/MWCNT and Bi/MWCNT nanocatalysts toward GA electrooxidation. Mass activities over the total potential for Au/MWCNT, Bi/MWCNT, and Au_80_Bi_20_/MWCNT were determined as 1024.60, 200.82, and 1601.64 mA/mg Au, respectively. These results were consistent with the results from CV, CA, and EIS.

Electrode preparation parameters for maximum glucose electrooxidation, namely V_c_, t_u_, and t_d_ were optimized by using RSM. In optimization studies, the working electrode was modified with the AuBi nanocatalyst. Table 2 exhibits the experimental design from CCD and corresponding response values. The obtained data depict that the GAEO on AuBi can be modeled well with the quadratic regression model at determined conditions. Accordingly, Eqn. 4 and Eqn. 5 depict model equations for GAEO consisting of real and coded factors, respectively.

Specific Activity = 1.01–0.12 A+0.14B–0.024 C–0.097AB+0.045AC+0.005BC+0.22A^2^–0.24B^2^–0.13AC^2^ (4)

Specific Activity = 0.79335–0.073089 V_c _+ 0.025536 t_u _+ 0.014417 t_d _-0.000455874 V_c_t_u _+0.000428062 V_c_t_d _– 0.0000116891 t_u_t_d _+ 0.00410766 V_c_^2 ^– 0.000280484 t_u_^2^–0.000613988 t_d_^2^ (5)

The adequacy and significance of the model were validated with analysis of variance (ANOVA). The ANOVA results depict that the P-value of the model is less than 0.05, and this indicates that the model is statistically significant (Table 5). Besides, the determination of coefficient and adequate precision values of the model were found to be 0.89 and 10.7, respectively. The fact that the lack of fit value was statistically insignificant indicates that the model depicts a good agreement with the experimental data. Accordingly, the proposed model can be utilized to navigate the design space [53]. 

**Table 5:** ANOVA regression model for GAEO.

Source	Sum of Squares	df	Mean Square	F-value	p-value	
Model	0.76	9	0.084	9.27	0.0009	significant
A-Volume of nanocatalyst mixture	0.15	1	0.15	16.11	0.0025	
B-Ultrasonification of nanocatalyst mixture	0.19	1	0.19	21.26	0.0010	
C-Duration of drying	0.00576	1	0.00576	0.63	0.4444	
AB	0.076	1	0.076	8.37	0.0160	
AC	0.016	1	0.016	1.78	0.2114	
BC	0.0002	1	0.0002	0.022	0.8850	
A²	0.13	1	0.13	14.11	0.0037	
B²	0.16	1	0.16	18.03	0.0017	
C²	0.046	1	0.046	5.04	0.0485	
Residual	0.091	10	0.009086			
Lack of Fit	0.057	5	0.011	1.71	0.2852	not significant
Pure error	0.034	5	0.006707			
Cor total	0.85	19				

Figure 8 depicts the response surface plots for t_u_, V_c_ and t_d_ parameters. The interaction between t_u_ and V_c_ for specific activity toward GAEO was presented in Figure 8a. Specific activity for GAEO decreases when the V_c_ value is increased from 0.5 to 7.75 µL. An increase in specific activity was observed for nanocatalyst loads higher than 7.75 µL. Figure 8b depicts that the specific activity increases up to about 15 min of t_d_ and begins to decrease after this maximum point. It was observed that the AuBi nanocatalyst could not attach enough to the electrode surface at very low t_d_ values, and some of the nanocatalysts were removed from the electrode surface. At higher t_d_ values, lower specific activities were observed as a result of oxidation of metals and vaporizing of Nafion in the nanocatalyst slurry. It was determined from Figure 8c that the relation of the specific activity with t_u_ depicts a volcano shape. The specific activity of AuBi for GAEO increased up to about 45 min of t_u_, and a decrease was observed after this value. This may be due to the sonification time affecting the crystal structures of the nanoparticles. Pollet et al. emphasized that during high sonification periods, the crystallinity of nanoparticles can be disrupted, and the formation of amorphous structures could be observed [54]. 

**Figure 7:**
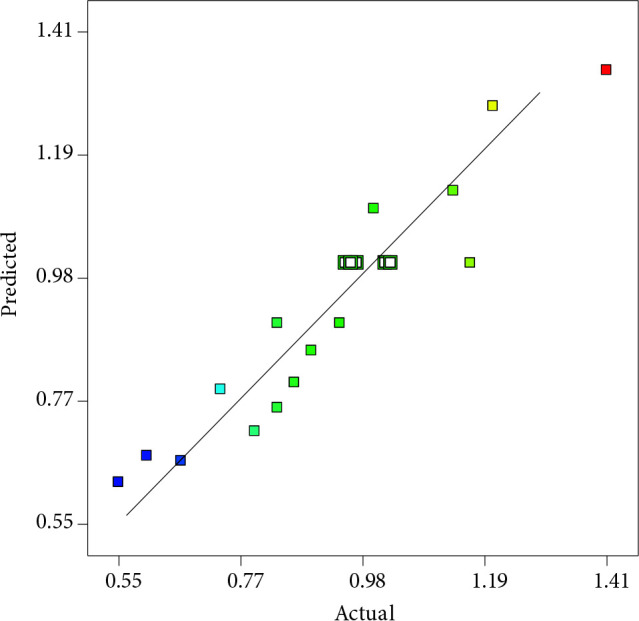
Comparison of experimental and predicted values for GAEO.

**Figure 8:**
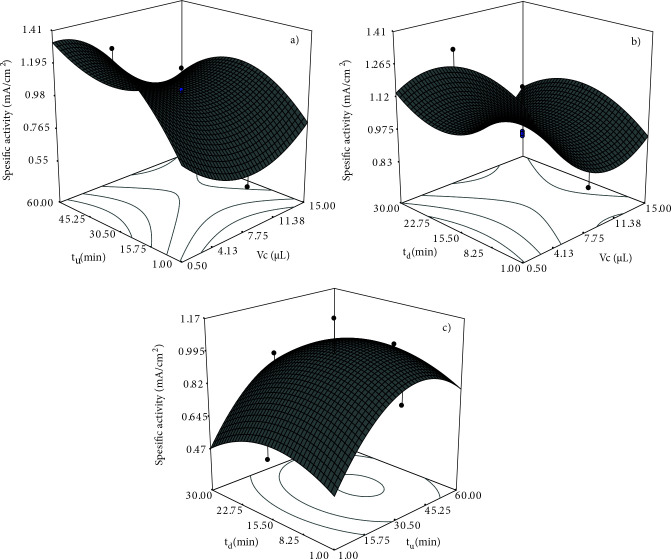
Response surface plots of a) t_u_ and V_c_, b) t_d_ and V_c_, and c) t_d_ and t_u_ for GAEO.

Design-Expert software was used to determine optimum conditions for GAEO, and related results were summarized in Table 6. The V_c_ of 0.5 µL, t_u_ of 44.87 min, and t_d_ of 11.49 min were obtained as an optimum condition for electrode preparation toward GAEO on AuBi/MWCNT. It could be seen in Table 6 that specific activity under optimum conditions was predicted by the obtained model as 1.40971 mA/cm^2^. The experiment was conducted under optimum conditions to verify the specific activity value derived from the model, and the specific activity was found to be 1.62 mA/cm^2^. It was determined that the obtained model was close to the experimental value with an error of 13%, indicating that the predicted value was in harmony with the observed value. 

**Table 6:** Optimum conditions by the CCD.

Number	Vc (µL)	tu (min)	td (min)	Spesific activity (mA/cm2)	Desirability
1	0.50	44.87	11.49	1.40971	1.000
2	0.50	42.63	12.83	1.40723	0.997
3	0.50	49.08	13.28	1.4027	0.992
4	0.50	46.53	14.81	1.40211	0.991
5	0.50	42.17	17.43	1.38618	0.972
6	15.00	32.98	16.67	1.10313	0.643
7	15.00	32.85	16.79	1.10311	0.643
8	15.00	33.83	17.28	1.10268	0.643

**Figure 9:**
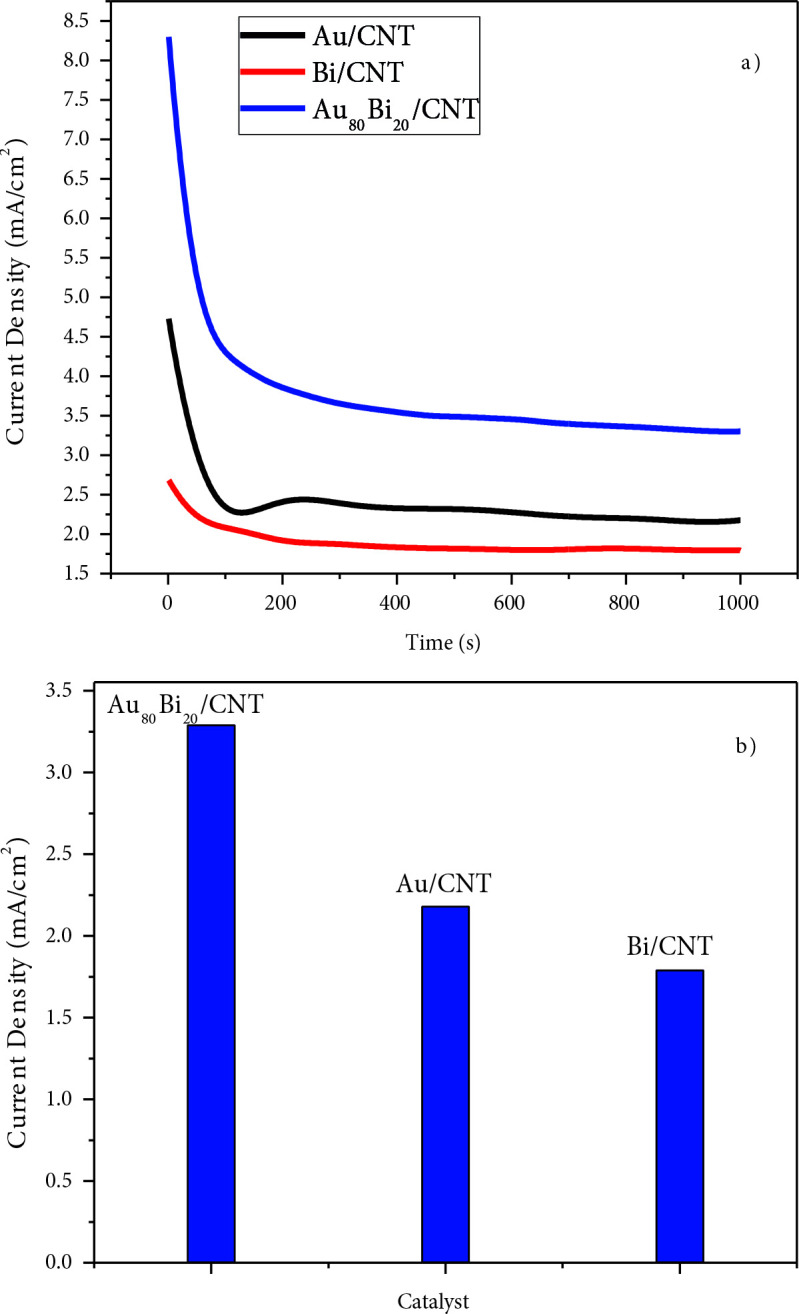
Chronoamperomograms of Au/MWCNT, Bi/MWCNT, and Au_80_Bi_20_/MWCNT in 0.5 M GA at -0.4 V.

The stability and GAEO activity of synthesized nanocatalysts were determined via CA. Figure 9a depicts CA results of Au/MWCNT, Bi/MWCNT, and Au_80_Bi_20_/MWCNT in 0.5 M GA at –0.4 V potential. Figure 9b depicts the currents at the end of 1000 s. At the end of 1000 s, Au_80_Bi_20_/MWCNT current value is approximately 1.4 and 1.8 times greater than Au/MWCNT and Bi/MWCNT, respectively. Moreover, Au_80_Bi_20_/MWCNT has the best stability and highest GAEO activity in the long term.

**Figure 10:**
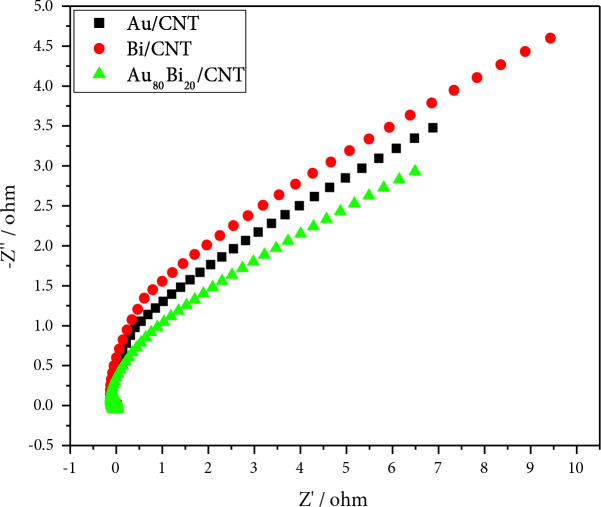
Nyquist plots obtained in 0.5 M GA for electrodes modified with Au/MWCNT, Bi/MWCNT, and Au_80_Bi_20_/MWCNT at –0.1 V.

Figure 10 depicts the Nyguist plot for Au/MWCNT, Bi/MWCNT, and AuBi/MWCNT in 0.5 M GA. The shape of the Nyguist plots is generally semicircle, and the diameter of these semicircles has a significant effect on the charge transfer resistance of catalyst. Accordingly, when the diameter of the semicircles decreases, the charge transfer resistance decreases and the GAEO activity of the nanocatalyst increases. According to Figure 10, the charge transfer resistance can be listed as Au_80_Bi_20_/MWCNT <Au/MWCNT <Bi/MWCNT. The fitted EIS profile of Au/MWCNT, Bi/MWCNT, and AuBi/MWCNT were given in Figure S1, Figure S2, and Figure S3, respectively. The charge transfer resistance of Au/MWCNT, Bi/MWCNT, and AuBi/MWCNT were determined as 2.502, 3.733, and 2.279 Ω, respectively. As a result, it was found that Au_80_Bi_20_/MWCNT nanocatalyst has the highest GAEO activity, and these results are in agreement with CV and CA results.

## 4. Conclusion

Au/MWCNT, Bi/MWCNT, and bimetallic Au-Bi/MWCNT were synthesized via NaBH_4_ reduction method, characterized by advanced surface analytical methods. The electrocatalytic performance of prepared catalyst was investigated with EIS, CV, CA, and LSV toward GAEO. Following results and insights were obtained: 

Ø Au/MWCNT, Bi/MWCNT, and Au-Bi/MWCNT at varying Au:Bi ratios could be easily prepared from corresponding Au and Bi precursors via NaBH_4_ reduction method.

Ø According to XRD and TEM results, particle sizes of Au_80_Bi_20_/MWCNT were compatible with each other. It was observed that the BET surface area of Au/MWCNT increased with the addition of Bi.

Ø Electrochemical measurement was revealed that Bi addition improves the electrochemical activity of Au/MWCNT. This situation can be explained by electronic effect.

Ø According to CV results, Au_80_Bi_20_/MWCNT showed the highest GAEO performance. The optimum metal molar ratio is the basis for this performance.

Ø CCD was utilized for optimum conditions of the electrode preparation. The volume of nanocatalyst slurry (V_c_, A), ultrasonication time of the nanocatalyst slurry (t_u_, B), and the drying time of the electrode (t_d_, C) are determined as independent variables. The maximum current density values obtained for GAEO were identified as the response. The V_c_ of 0.5 µL, t_u_ of 44.87 min, and t_d_ of 11.49 min were obtained as an optimum condition for electrode preparation toward GAEO on AuBi/MWCNT. 

Ø CA and EIS results revealed that AuBi nanocatalyst has a high stability and fast oxidation kinetics. 

Ø The data obtained from this study depicts that Au_80_Bi_20_/MWCNT nanocatalyst is a good candidate as anode nanocatalyst for DGFC. 
